# Developing a Cross-National Disability Measure for Older Adult Populations across Korea, China, and Japan

**DOI:** 10.3390/ijerph191610338

**Published:** 2022-08-19

**Authors:** Sanghun Nam, Mi Jung Lee, Ickpyo Hong

**Affiliations:** 1 Department of Occupational Therapy, Graduate School, Yonsei University, Wonju 26493, Korea; 2 Department of Nutrition, Metabolism, and Rehabilitation Sciences, University of Texas Medical Branch, Galveston, TX 77555, USA; 3 Department of Occupational Therapy, College of Software and Digital Healthcare Convergence, Yonsei University, Wonju 26493, Korea

**Keywords:** cognitive function, physical function, cross-national, East Asian older adults

## Abstract

This study aims to develop a universal scale for comparing cognitive and physical functions among countries using health survey data from China, Korea, and Japan. This study used the data of 934 participants from the Korean Longitude Study of Aging, 2506 participants from the China Health and Retirement Longitude Study, and 178 participants from the Japanese Study of Aging and Retirement. Each physical and cognitive function item in the three countries has five key items. The anchoring method used the key items to link each cognitive and physical function of the three countries. We investigated the psychometric characteristics of the final item using the Rasch analysis. We extracted 13 items of 19 cognitive function items and 20 items out of 29 physical function items using the anchoring method and the Rasch analysis. The Rasch analysis showed good fit statistics for 13 cognitive function items and 20 physical function items. The measurement scale developed in this study will enable valid comparisons of older adults’ cognitive and physical functions across these three countries.

## 1. Introduction

The global population over 65 years old is projected to reach 1.6 billion by 2050 [1]. In Korea, the population of 65 years old or older accounted for 15.7% of the total population in 2020, and households with Koreans aged 65 years or older accounted for 22.8% of the total households [2,3]. The number of older adult households in Korea is expected to account for 49.6% in 2047. The aging problem is emphasized because aging accompanies various diseases, such as dementia and chronic diseases [4,5,6]. The mortality rate among older adults in Korea due to chronic diseases reached 79.9% [7]. Additionally, the prevalence of dementia as of 2018 is as follows: 1% aged 65–69 years, 4% aged 70–74 years, 21% aged 75–79 years, and 40% aged 85 years or older [8]. Stites, Karlawish [9] investigated its effects on the self-reported quality of life (QOL) in individuals with various cognitive impairments (normal cognition, mild cognitive impairment, and mild Alzheimer’s disease dementia). Hence, individuals with mild cognitive impairment and mild Alzheimer’s disease dementia reported lower QOL than individuals with normal cognition despite controlling for sex [9]. Korea, China, and Japan reported increasing medical expenses and burden on caregivers due to aging and deterioration of various cognitive and physical functions [10,11,12].

Several studies have conducted international health comparisons of older adults. These studies provide an opportunity to compare public health across countries and establish appropriate countermeasures for aging [13]. Díaz-Venegas, Reistetter [14] reported disability progression using the Mexican Health and Aging Study (MHAS), including the 2001 baseline survey and 2003 follow-up survey, and the Health and Retirement Study (HRS), including the 2000 and 2002 surveys. Both data reported that approximately 44% of the samples maintained the same disability level during the 2-year follow-up period. Additionally, MHAS showed differences in disability progression according to sex; however, HRS consistently showed and reported disability progression in both males and females [14]. The Hong, Reistetter [15] study used the Rasch analysis to create metrics and to compare and analyze health in American and Mexican geriatric populations and revealed that adults in the United States performed worse than adults in Mexico (β = −0.26) and two chronic diseases (arthritis, β = −0.36; lung problems, β = −0.62) [15]. Additionally, a cross-country comparative study on factors related to cognitive function in the United States and Korea reported that depression, hypertension, diabetes, and alcohol consumption as factors related to cognitive function in the United States [16]. In Korea, marital status and hearing impairment were reported as factors related to cognitive function [16]. Hong, Simpson [17] developed the same measurement framework using the Rasch common-item equating method and compared the average disability levels in the United States and Korea and reported that the disability level between the two countries was lower in the United States than in Korea. The Rasch common-item equating method used by Hong, Simpson [17] is one of the methods that can resolve the measurement discrepancy between items in both countries. However, there should be no difference between the living environment and culture to compare the health of older adults between countries. These cultural differences between countries can differently interpret the concept of health [18].

Korea, China, and Japan are adjacent countries and have common social and cultural characteristics [19,20]. Korea and Japan have almost the same demographic and economic development structures, and identical racial similarities and diseases caused by similar diets. Likewise, China entered the period of an aging society in 2001, and Korea entered the same period in 2000; however, Japan entered an aging society in 1970. Japan achieved considerable progress in health and community policies for older adults corresponding to their rapidly aging population [21]. Japan’s major welfare policies for older adults include a policy of free medical expenses for older adults (1973), enactment of the Elderly Health Act (1982), and the Gold Plan (1994). Additionally, attention is paid to the fact that Japan’s welfare needs for older adults mainly include living problems centered on recovery. Accordingly, a long-term insurance system was introduced in April 2000 to supplement the existing welfare issues of older adults, and the older adults’ medical system is the core of Japan’s welfare policy for older adults [22]. Therefore, China, Korea, and Japan have already held six aging meetings from 2010 to 2016, shared the current state of aging policies, established a cooperative system to respond to aging, and shared experiences [23].

Our previous studies summarized seven studies that compared health in three countries using data from China, Korea, and Japan [24], and firstly revealed that a study on cognitive function after retirement in three countries showed that all three countries had a declining cognitive function after retirement according to sex, educational attachment, and wealth level. In this study, word recall was used to measure cognitive function. China and Japan used ten words and Korea used 3 words, which revealed limitations on item inconsistency. Secondly, the comparison of the proportion of successful aging between China and Korea revealed that Korea was 25.2%, which was higher than China with 18.6%. Successful aging refers to gradual physiological and functional change adaptation over time, while experiencing mental attachment and the meaning and purpose of life [25]. Finally, Japan (29.2%) reported the highest rate, and China (15.7%) the lowest when successful aging was compared among the three countries. The literature review revealed a lack of comparison items as a common difficulty in these cross-country health comparisons. The three countries lack the same items despite much data. Additionally, the data on older adults that each country collects may differ from each country’s item because it contains cultural aspects [26,27,28].

This study aims to link the health survey data items of the three countries before comparing cognitive and physical functions across Korea, China, and Japan. Linking of measurement scale using data across the three countries uses the “anchoring method” [29,30]. Our study will be the first step in possible comparison of cognitive and physical functions between Korea, China, and Japan.

## 2. Materials and Methods

### 2.1. Study Data

Funded by the Ministry of Labor, the Korean Longitudinal Study of Aging (KLoSA) is the first national survey of Korean aging and is available to the public. KLoSA was designed to compile information on various aging aspects for basic statistical data in interdisciplinary research on social, economic, physical, and psychological aging aspects. KLoSA, based on a nationally representative sample of Koreans aged 45 years or older, was launched in 2006 and collected every 2 years [31]. More information about KLoSA is found on the website (https://survey.keis.or.kr (accessed on 29 May 2022)).

The China Health and Retirement Longitudinal Study (CHARLS) is funded by Peking University (China), the World Bank, and the National Institute on Aging (China). The following are indicators contained in the CHARLS questionnaire: population structure and layout, family composition, health situation and health service utilization, working situation and insurance benefits, household consumption level, assets situation, etc. CHARLS is a longitudinal questionnaire of national representativeness for adults over the age of 45 years, covering their social, economic, and health situations; it was launched in 2011, and data is collected every 2 years [32]. Visit the website for more information (http://charls.pku.edu.cn/index/en.html (accessed on 29 May 2022)).

The Japanese Study of Aging and Retirement (JSTAR) is supported by a collaboration between the Research Institute of Economy, Trade, and Industry, and Hitotsubashi University. JSTAR collected various information, such as income, wealth, assets, and health, as an interdisciplinary data resource on Japan’s health, economic status, and QOL. JSATR data collection was conducted every 2 years from 2007 to 2013 for adults aged 50 years and older [33]. More information can be found on the website (https://www.rieti.go.jp/en/projects/jstar/ (accessed on 29 May 2022)).

We selected individuals with stroke and heart disease from KLoSA, CHARLS, and JSTAR data. An individual’s disease can significantly impact cognitive and physical functions. Their cognitive and physical functional status are similar because stroke and heart disease are homogeneous with each other. Therefore, this study focused on individuals with stroke and heart disease.

### 2.2. Cognitive Function and Physical Function Item Extraction

Our previous study, Delphi, identified a total of 15, 7, and 11 items for cognitive function (Table 1) and 17, 19, and 15 items for physical function (Table 2) from KLoSA, CHALRS, and JSTAR, respectively, for cross-international comparison, based on the international classification of functioning [34]. Items with the same contents were selected for key items to link items across three datasets. For example, “bathing or showering” in KLoSA, “have any difficulty with bathing or showering” in CHALS, and “bathing on their own” in JSTAR indicates bathing in the house. Therefore, we selected five for cognitive and five for physical function for our key items. The total cognitive function items in Korea, China, and Japan is 19, excluding the key items and items that overlap in more than 1 country. Of the total physical function items, 29 were found in Korea, China, and Japan.

### 2.3. Statistical Analysis

**Figure 1:**
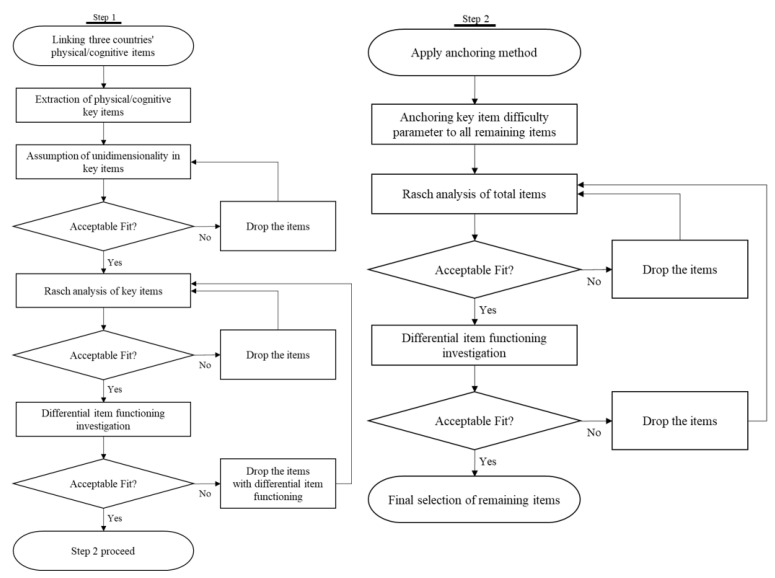
Flow chart of this study.

This study followed a two-step process to anchor items from three countries. Step one is confirmatory factor analysis (CFA) to test the unidimensionality assumption of the selected key items (orientation time, orientation-week, subtraction, verbal memory input, and verbal memory output in cognitive and dressing, bathing, eating, get in/out of bed, and toileting in physical). Then, we conducted the Rasch analysis to investigate the key items’ fit and difficulty, as well as differential item functioning (DIF). Step two is the use of the anchoring method to link three measures through key items. The Rasch analysis was conducted on all linked items to validate the contents of item difficulties and DIF (Figure 1). This study preprocessed data and performed descriptive statistics from KLoSA, CHARLS, and JSTAR using SAS version 9.4 (SAS Institute, Cary, NC) and CFA analysis and anchoring process with Mplus version 8.4 (Los Angeles, CA, USA) and Winsteps version 5.2 (Portland, OR, USA).

### 2.4. Step One: Test the Psychometric Properties of Key Items

#### 2.4.1. Unidimensional Assumptions about Key Items

CFA analysis was performed to test the unidimensionality assumptions of key items. We used the criteria of comparative fit index (CFI), Tucker–Lewis index (TLI), Root mean square error of approximation (RMSEA), and standardized root mean square residual in CFA analysis [35]. Values above 0.95 indicate a good fit, while 0.90 and <0.95 are considered a marginally acceptable good fit for CFI and TLI. A value close to 0.06 indicates a good fit, a value between 0.06 and 0.08 indicates a moderate fit, a value <1.00 indicates an acceptable fit, and a value >0.10 indicates a poor fit for RMSEA. The acceptable SRMR value is <0.5 [36] (pp. 136–162) [37,38,39]. We investigated the local independence assumption by ensuring that the mean of all residual correlations for a key item is <0.20.

#### 2.4.2. Rasch Analysis for Key Items

After investigating the CFA, we used the Rasch model to investigate the item fit statistics of key items [40,41]. The criteria of infit and outfit mean-square residuals (MnSq, 0.60–1.60) and z-standardized (ZSTD, −2.0–2.0) were used for the Rasch analysis [42,43]. We removed misfit items through the Rasch analysis.

#### 2.4.3. DIF for Key Items

DIF for sex and age was tested using the generalized Mantel–Haenszel test [44]. A significant DIF was removed if each item exhibited a *p*-value of <0.05 at a DIF contrast value of >0.43 [29]. We evaluated person invariance for the items with DIF.

### 2.5. Step Two: Anchoring the Difficulty Parameters of Key Items to the Entire Database

#### 2.5.1. Anchoring Methods

In step two, the total cognitive and physical function items are linked by the anchoring method, using the key items analyzed in step one. We anchored the difficulty parameters of the key items to the total items of each of the cognitive and physical functions using Winsteps software [29]. The second step will generate the unified total cognitive and physical function measures across the three countries using the anchoring method.

#### 2.5.2. Rasch Analysis for the Total Cognitive and Physical Function Measures

The inclusion of misfit items in the total cognitive and physical function measures generated in this step by Rasch analysis was investigated. The total cognitive and physical function measures selected in step two removed misfit items step by step through the Rasch analysis. The criteria for the fit statistics are the same as those of the Rasch analysis performed in step one.

#### 2.5.3. DIF for the Total Cognitive and Physical Function Measures

Finally, the DIF was investigated for the total cognitive and physical function measures that are finally selected in step two. The DIF according to sex and age was analyzed using the generalized Mantel–Haenszel test [44]. We removed items with a *p*-value of <0.05 at a DIF contrast value of >0.43. We evaluated person invariance for the items with detected DIF.

## 3. Results

KLoSA participants were older (70.07 years, SD = 8.92) than CHALRS (62.47 years, SD = 10.04) and JSTAR (63.38 years, SD = 6.22) on average. Female participants were dominant in KLoSA (55.03%) and CHARLS (58.29%), but not in JSTAR (44.38%). For the proportion of educational achievement, most participants in KLoSA (72.70%) and CHALRS (86.30%) graduated from middle school (or from schools at a lower educational level), except for JSTAR (12.43%). The least reported marital status was single in all three countries (KLoSA, 27.30%; CHALRS, 16.69%; and JSTAR, 24.16%). The highest reported self-rated health was JSTAR (3.08, SD = 1.09) among the three datasets, and the lowest reported self-rated health was CHARLS (3.73, SD = 0.91). Finally, the country with the highest rate of heart disease was China (83.52%), and the country with the highest stroke rate was Korea (n = 360, 38.54%).

### 3.1. Step One: Test the Psychometric Properties of Key Items

#### 3.1.1. Unidimensional Assumptions about Key Items

A unidimensionality assumptions test was conducted for the five key items of cognitive and physical function items in the database of the three countries using the CFA. CFA explained the good model fit values for key physical function items (RMSEA = 0.028; CFI = 1.000; TLI = 1.000; SRMR = 0.01). Among the five key items of cognitive function, the “oriented week” item was removed for exceeding 0.2 of a residual correlation. For the four key items, CFA showed a moderate fit value (RMSEA = 0.096; CFI = 0.974; TLI = 0.921; SRMR = 0.04).

#### 3.1.2. Rasch Analysis for Key Items

The Rasch analysis demonstrated an acceptable fit of all the key items of cognitive and physical functions (Table 3).

#### 3.1.3. DIF for Key Items

DIF in bathing (DIF contrast = −0.76, *p* < 0.05)/toileting (DIF contrast = 1.13, *p* < 0.05) items of the physical function and the subtraction (DIF contrast = 0.71, *p* < 0.05)/verbal memory input (DIF contrast = −0.53, *p* < 0.05) items of the cognitive function were detected. A cross-plot with a 95% confidence interval (CI) for the person measures with and without DIF items of cognitive and physical function indicated that the person measure parameters were not affected by the DIF items of cognitive and physical functions. Therefore, cognitive and physical functions that are not eliminated DIF items included bathing, toileting, subtraction, and verbal memory input items.

### 3.2. Step Two: Anchoring the Difficulty Parameters of Key Items to the Entire Database

#### 3.2.1. Anchoring Methods

The difficulty parameters of five key physical function items and four key cognitive function items that are analyzed in step one were anchored to the total items using the anchoring method. Among the 29 items in which the difficulty parameter of the key item of physical function was anchored, the easiest item was “eating” (logit = −1.57), and the most challenging item was “walking 100 m” (logit = 5.96). Among the 19 items for which the difficulty parameter of the key items of cognitive function was anchored, the easiest item was “How to use objects 1 (logit = −5.06),” and the most challenging item was “calculations for percentages 2 (logit = 3.06).”

#### 3.2.2. Rasch Analysis for the Total Cognitive and Physical Function Measures

Rasch analysis for the total cognitive and physical function measures.

Rasch analysis revealed that 29 anchored items of physical functions fit the Rasch model. We removed the “calculations for percentages 2” item from the 19 anchored items of cognitive function, as it showed misfit statistics in the Rasch analysis (infit MnSq = 1.58, infit ZSTD = 3.77, outfit MnSq = 3.26, outfit ZSTD = 6.28). Through Rasch analysis, 29 physical function items and 18 cognitive function items were selected.

#### 3.2.3. DIF for the Total Cognitive and Physical Function Measures

DIF for the total cognitive and physical function measures.

The Rasch analysis of physical function items identified 11 items with DIF. However, the items of “laundry” and “Going out using public transport” converged without being removed because there was no measured value for the intermediate items. Among the 20 physical function items, the easiest item was “eating” (logit = −1.57), and the most difficult item was “running/jogging for 1 mile” (logit = 5.36).

The Rasch analysis of cognitive function items identified 5 DIF items, including orientation-week, calculations for percentages 1, 3, and 4, or enforcement 3 (write about mood or weather) and were thus removed. Of the 13 selected items, the easiest item was orientation time, and the most difficult item was enforcement 1 (turn the paper upside down, fold it, and hand it over) (Table 4).

## 4. Discussion

This study developed a measurement scale by linking each cognitive and physical function item in Korea, China, and Japan. The result revealed that 13 cognitive and 20 physical function items were selected. Several studies using data from each country limited the number of common items because they tried to identify and compare similar items. However, the item work to link in this study assimilated the scale of all remaining items through key items. Therefore, the items selected in this study will help measure universally and compare cognitive and physical functions across Korea, China, and Japan.

The key item anchoring method using Winsteps in this study included the linkage of the interval scale achieved by anchoring the parameter estimates of the key item to the total cognitive and physical function items [29,30]. The Winsteps program also employed joint maximum likelihood estimation (JMLE) to calculate parameter estimates [45,46,47]. JMLE had some limitations in calculating these parameter estimates. First, the estimate of the item parameters could not be a consistent estimator if the number of items was fixed and the number of respondents indefinitely increased. The parameter estimate did not become a consistent estimator, although the number of respondents was fixed and the number of items indefinitely increased [48,49]. Second, the calculation difficulty considerably increased as the number of respondents increased, and the ability of the respondents who answered all the questions and those who failed even one question cannot be estimated [48,49]. Therefore, this study solved the problem using conditional maximum likelihood estimation in which the raw score was a sufficient statistic for the ability parameter as an estimation method based on conditional probability [50].

This study used the five key items (dressing, bathing, eating, getting in/out of bed, and toileting) to link physical function items. The scale of physical functions using the five key items was 20 items. The 20 items consisted of various items that are used for general activities of daily living. Examples include sitting in a chair for 2 h, reaching out, pushing/pulling objects, climbing stairs, running, jogging, and picking up small objects. These items are appropriate for evaluating physical functions because they can examine the upper and lower extremity functions and basic daily activities [51,52]. However, items that are associated with cultural differences may exist. Particularly, the item “sitting in a chair for 2 h” may be inappropriate for older adults who lead a sedentary life [53,54]. Therefore, researchers should pay attention to this item when measuring body functions or performing cross-country comparisons.

In this study, the items “laundry” and “going out using public transport” should have been removed by DIF in the work to link physical functions but were included because there was no intermediate item measure value. The cross-plot of the 95% CI for person measures with and without DIF items showed that the person measure parameters were not influenced by the two DIF items although 2 out of 20 items showed DIF (laundry, going out using public transport) [30]. The person measure value was 4.44 before deleting the 2 items and 3.63 after deleting the 2 items. The individual’s ability did not decrease but increased if these two items were not deleted.

We linked the cognitive function scale using the difficulty parameter of 4 key items and revealed a total of 13 items. Four calculations for percentages were removed before the final selection. The model fit was not good in the Rasch analysis for the problem with calculation difficulty because it was related to older adult data. Complex arithmetic calculations may be impossible for most seniors as individuals grow older [55,56,57]. Therefore, the final 13 items from which calculation items were removed were considered appropriate for comparing cognitive functions between the three countries.

This study had several limitations. First, there is the issue regarding the data collection period used to link items from the three countries. We used data from 2011 for China and Japan and 2010 for Korea. A large difference can be observed in the data collection period compared with 2022. In the case of JSTAR, the most recent data we could access dated back to 2013. Therefore, future research needs to use data with a collection period close to the current.

## 5. Conclusions

This study developed a measurement scale that can compare the cognitive and physical functions of older adults using data from China, Japan, and Korea. These results will provide a platform for sharing health policies, treatment methods, and intervention approaches by comparing the health of neighboring East Asian countries as population aging continues to progress.

## Figures and Tables

**Table 1:** Cognitive function items in the 2011 CHARLS, 2011 JSTAR, and 2010 KLoSA.

No.	Type	CHARLS	JSTAR	KLoSA
1	Key I	Orientation time	Orientation time	Orientation time
2	Key I	Orientation-week	Orientation-week	Orientation-week
3	Key I	Subtraction	Subtraction	Subtraction
4	Key I	Verbal memory input	Verbal memory input	Verbal memory input
5	Key I	Verbal memory output	Verbal memory output	Verbal memory output
6	CK	Orientation-season	-	Orientation-season
7	JK	-	Orientation-location	Orientation-location
8	JK	-	Orientation-address	Orientation-address
9	K	-	-	How use of stuff-1
10	K	-	-	How use of stuff-2
11	K	-	-	Repeat speaking
12	K	-	-	Enforcement-1
13	K	-	-	Enforcement-2
14	K	-	-	Enforcement-3
15	CK	Enforcement-4		Enforcement-4
16	J	-	Percentage calculation-1	-
17	J	-	Percentage calculation-2	-
18	J	-	Percentage calculation-3	-
19	J	-	Percentage calculation-4	-

Note: Key I: Key Items; CK: Common items between China and Korea; JK: Common items between Japan and Korea; K: Korea items; J: Japan items.

**Table 2:** ADL and IADL function items in the 2011 CHARLS, 2011 JSTAR, and 2010 KLoSA.

No.	Type	CHARLS	JSTAR	KLoSA
1	Key I	Dressing	Dressing	Dressing
2	Key I	Bathing	Bathing	Bathing
3	Key I	Eating	Eating	Eating
4	Key I	Get in/out of bed	Get in/out of bed	Get in/out of bed
5	Key I	Toileting	Toileting	Toileting
6	CK	Urination control	-	Urination control
7	CK	Household chores	-	Household chores
8	CK	Preparing hot meals	-	Preparing hot meals
9	CK	Shopping	-	Shopping
10	CK	Managing assets	-	Managing assets
11	CK	Taking medications	-	Taking medications
12	C	Running/jogging 1 mile		-
13	C	Walking 1 mile	-	-
14	CJ	Walking 1 block	Walking 1 block	-
15	CJ	Getting up from a chair	Getting up from a chair	-
16	CJ	Climbing flights of stairs	Climbing flights of stairs	-
17	CJ	Reaching arms	Reaching arms	-
18	CJ	Lifting weights (10 lb.)	Lifting weights (10 lb.)	-
19	CJ	Picking up a coin	Picking up a coin	-
20	J	-	Walking around in the room	-
21	J	-	Sitting in a chair for 2 h	-
22	J	-	Climb one stair without handrail	-
23	J	-	Pushing or pulling large objects	-
24	K	-	-	Washing teeth & hair
25	K	-	-	Grooming
26	K	-	-	Laundry
27	K	-	-	Going out using public transport
28	K	-	-	Going out without public transport
29	K	-	-	Receive calls

Note: Key I: Key Items; CK: Common items between China and Korea; C: China items; CJ: Common items between China and Japan; J: Japan items; K: Korea items.

**Table 3:** ADL and IADL function items in the 2011 CHARLS, 2011 JSTAR, and 2010 KLoSA.

Key Items	CMLE Measure (Logits)	Model SE	Infit		Outfit		DIF Contrast	Mantel–Haenszel Probability
			MnSq	ZSTD	MnSq	ZSTD		
Physical functions								
Dressing	−0.06	0.14	1.01	0.16	0.97	−0.25	−0.24	0.5368
Bathing	1.62	0.13	0.93	−1.41	0.87	−1.00	−0.76	0.0056
Eating	−1.57	0.19	1.02	0.23	1.03	0.18	−0.39	0.5004
Getting in/out of bed	−0.66	0.15	0.80	−2.34	0.74	−1.82	−0.07	0.6977
Toileting	0.67	0.13	1.24	3.54	1.34	3.52	1.13	0.0001
Cognition functions								
Orientation time	−0.64	0.06	1.09	3.19	1.11	2.67	0.26	0.0408
Subtraction	0.75	0.05	1.12	5.51	1.15	4.26	0.71	0.0000
Verbal memory input	−0.13	0.05	0.87	−5.70	0.85	−5.78	−0.53	0.0000
Verbal memory output	0.02	0.05	0.91	−4.16	0.90	−4.16	−0.37	0.0005

Note: CMLE: conditional maximum likelihood estimation; SE: standard error; MnSq: mean-square; ZSTD: Zstandard; DIF: differential item functioning; KLoSA: Korean Longitudinal Study of Aging; CHARLS: China Health and Retirement Longitudinal Study; JSTAR: Japanese Study of Aging and Retirement.

**Table 4:** Fit statistics of the physical and cognitive function of linking items for the 2011 CHARLS, 2011 JSTAR, and 2010 KLoSA.

Items	CMLE MEASURE (Logits)	Model SE	Infit		Outfit		DIF Contrast	Mantel–Haenszel Probability
			MnSq	ZSTD	MnSq	ZSTD		
Physical functions								
Running/jogging for 1 mile	5.36	0.07	1.04	1.01	1.42	3.58	0.18	0.0837
Laundry	3.39	0.20	1.30	3.53	1.04	0.24	−2.42	0.0000
Climbing stairs	3.39	0.06	0.70	−9.90	0.58	−7.41	0.23	0.2907
Walking for 1 mile	3.27	0.06	1.32	8.50	1.68	8.43	−0.26	0.2804
Going out using public transport	3.18	0.19	0.89	−1.27	0.98	0.08	1.96	0.0000
Physical functions								
Climbing one stair	1.79	0.37	1.02	0.20	0.75	−0.60	0.46	0.6321
Pushing or pulling large objects	1.68	0.37	0.79	−1.26	0.68	−0.84	0.28	0.8665
Shopping	1.65	0.08	0.86	−3.01	0.58	−4.43	0.07	0.7019
Reaching arms	1.24	0.09	1.20	3.46	0.90	−0.64	−0.04	0.7922
Sitting in a chair for 2 h	0.87	0.42	1.08	0.40	1.25	0.68	−0.38	0.9573
Getting up from a chair	0.87	0.10	1.05	0.77	0.65	−2.18	0.09	0.8987
Grooming	0.51	0.24	0.74	−1.99	0.59	−1.44	0.00	0.8927
Walking around in the room	0.34	0.47	1.17	0.64	0.80	−0.20	−0.51	0.5986
Receive calls	0.30	0.24	1.38	2.42	1.39	1.13	0.66	0.4982
Dressing	−0.06	0.11	0.80	−2.91	0.41	−2.85	−0.17	0.1773
Washing teeth & hair	−0.08	0.25	0.82	−1.26	0.73	−0.59	−0.54	0.5542
Picking up a coin	−0.18	0.12	1.10	1.09	0.65	−1.33	−0.22	0.7501
Urination control	−0.60	0.13	1.20	2.20	1.52	1.99	−0.24	0.3677
Get in/out of bed	−0.66	0.13	0.67	−4.40	0.22	−5.05	0.05	0.9619
Eating	−1.57	0.16	1.05	0.51	0.37	−3.57	0.02	0.6911
Cognitive functions								
Enforcement 1	0.87	0.09	0.83	−4.34	0.74	−2.10	−0.09	0.5060
Subtraction	0.75	0.05	1.13	5.86	1.21	3.29	0.56	0.0000
Verbal memory output	−0.02	0.05	0.97	−1.13	1.09	1.80	−0.43	0.0001
Enforcement 2	−0.05	0.09	0.85	−3.53	0.76	−2.30	0.05	0.9590
Verbal memory input	−0.13	0.05	0.94	−2.54	0.91	−1.97	−0.57	0.0000
Enforcement 4	−0.47	0.05	0.99	−0.29	1.16	3.09	0.31	0.0112
Orientation time	−0.64	0.05	1.03	1.15	1.05	0.92	0.15	0.1931
Orientation-address	−0.97	0.10	0.98	−0.33	1.54	4.31	0.22	0.6154
Orientation-season	−3.03	0.10	1.15	2.02	1.27	1.67	−0.21	0.6189
Repeat speaking	−3.16	0.16	0.81	−1.71	0.50	−1.95	0.49	0.5002
How to use objects 2	−5.15	0.30	0.88	−0.49	1.22	0.63	0.56	0.3756
Orientation-location	−5.25	0.30	0.82	−0.77	0.22	−2.84	−1.11	0.1655
How to use objects 1	−5.31	0.31	0.86	−0.57	0.95	0.01	0.69	0.2715

Note: CMLE: conditional maximum likelihood estimation; SE: standard error; MnSq: mean-square; ZSTD: Zstandard; DIF: differential item functioning; KLoSA: Korean Longitudinal Study of Aging; CHARLS: China Health and Retirement Longitudinal Study; JSTAR: Japanese Study of Aging and Retirement.

## Data Availability

Not applicable.
